# Phylogeographic analysis of hemorrhagic fever with renal syndrome patients using multiplex PCR-based next generation sequencing

**DOI:** 10.1038/srep26017

**Published:** 2016-05-25

**Authors:** Won-Keun Kim, Jeong-Ah Kim, Dong Hyun Song, Daesang Lee, Yong Chul Kim, Sook-Young Lee, Seung-Ho Lee, Jin Sun No, Ji Hye Kim, Jeong Hoon Kho, Se Hun Gu, Seong Tae Jeong, Michael Wiley, Heung-Chul Kim, Terry A. Klein, Gustavo Palacios, Jin-Won Song

**Affiliations:** 1 Department of Microbiology, College of Medicine, Korea University, Seoul, 02841, Republic of Korea; 2 The 5th R&D Institute, Agency for Defense Development, Yuseong P.O. Box 35, Daejeon, 34186, Republic of Korea; 3 The Armed Forces Medical Center, Saemaeul-ro, 177 beon-gil, Seongnam-si, Gyeonggi-do, 13574, Republic of Korea; 4 The Center for Genome Science, US Army Medical Research Institute of Infectious Disease at Fort Detrick, MD, 21702, USA; 5 5th Medical Detachment, 168th Multifunctional Medical Battalion, 65th Medical Brigade, Unit 15247, APO AP 96205-5247, United States of America; 6 Public Health Command District-Korea (Provisional), 65th Medical Brigade, Unit 15281, APO AP 96205-5281, United States of America

## Abstract

Emerging and re-emerging infectious diseases caused by RNA viruses pose a critical public health threat. Next generation sequencing (NGS) is a powerful technology to define genomic sequences of the viruses. Of particular interest is the use of whole genome sequencing (WGS) to perform phylogeographic analysis, that allows the detection and tracking of the emergence of viral infections. Hantaviruses, *Bunyaviridae*, cause hemorrhagic fever with renal syndrome (HFRS) and hantavirus pulmonary syndrome (HPS) in humans. We propose to use WGS for the phylogeographic analysis of human hantavirus infections. A novel multiplex PCR-based NGS was developed to gather whole genome sequences of Hantaan virus (HTNV) from HFRS patients and rodent hosts in endemic areas. The obtained genomes were described for the spatial and temporal links between cases and their sources. Phylogenetic analyses demonstrated geographic clustering of HTNV strains from clinical specimens with the HTNV strains circulating in rodents, suggesting the most likely site and time of infection. Recombination analysis demonstrated a genome organization compatible with recombination of the HTNV S segment. The multiplex PCR-based NGS is useful and robust to acquire viral genomic sequences and may provide important ways to define the phylogeographical association and molecular evolution of hantaviruses.

Defining viral genomic sequences are critical for the diagnosis, developing risk analyses and control strategies for emerging infectious diseases^1^. Recently, viral outbreaks of emerging pathogens are serious public health threats worldwide, in part as a result of lack of specific and rapid medical treatments^2^,^3^. Acquisition of whole virus genomic sequences based on next generation sequencing (NGS) increases the potential to clarify the etiology of infectious diseases and therapeutics^4^. NGS technology provides a robust tool to obtain quantitative genomic information using high throughput sequencing^5^. It is less-costly, highly efficient, and effective for the recovery of genomic sequences of clinical and environment samples. Currently, the technology applies to investigation for a variety of human diseases^6^,^7^,^8^. In addition, it promotes the understanding of microbes in humans leading for the control of metabolism and infectious diseases^9^,^10^. It has slowly advanced in the field of virology because of ultra-low copy and frequent variation of viral genomes obtained from specimens. However, the characterization of viral genomic sequences by NGS provides broad and deep insights into preventive and therapeutic strategies for the virus infections^11^.

Hantaviruses, family *Bunyaviridae*, are negative sense RNA viruses and contain tripartite RNA genomes; large (L), medium (M), and small (S) segments encoding RNA-dependent-RNA polymerase (L), membrane glycoproteins (Gn and Gc), and nucleocapsid protein (N), respectively^12^. Hantaviruses are the causative agents of hemorrhagic fever with renal syndrome (HFRS) in Eurasia and hantavirus pulmonary syndrome (HPS) in the Americas^13^,^14^,^15^. They are a significant public health threat with ~200,000 clinical cases reported annually and a fatality rate ranging from 1 to 35% worldwide^16^. Hantaviruses are transmitted to human via the respiratory route of aerosolized infectious particles from rodent excreta or bite of infected rodents. *Apodemus agrarius* is the reservoir of Hantaan virus (HTNV), an etiological agent of HFRS in the Republic of Korea (ROK) and China^17^. Common symptoms of HFRS include headache, myalgia, abdominal and back pain, nausea, vomiting, and diarrhea. The typical disease course consists of five phases; febrile, hypotensive, oliguric, diuretic, and convalescent^18^. The febrile phase is an initial stage defined by fever, pains, and edema during 3–5 days. The hypotensive phase lasts few hours and identified as decreased blood pressure, internal bleeding, proteinuria, and thrombocytopenia. The oliguric phase lasts for 3–7 days and is marked by renal dysfunction (reduced urine output), hypervolemia, and blood electrolyte imbalance. The diuretic (increased urine output) and convalescent phases are recovery stages that last for several weeks to months, and is characterized by progressive improvements in glomerular filtration rate, renal blood flow, and urine output control. A critical hallmark of HFRS is capillary leakage that results in edema and hemorrhage, suggesting that the vascular endothelium is damaged by cytokine storm against HTNV infection^19^. Hantavirus infections remain less understandable due to the lack of animal experimental models, difficulties in propagating the agent, and reverse genetic tools.

In this study, a method based on multiplex PCR-based NGS to achieve whole genome sequencing of HTNV L, M, and S segments from human or mammalian clinical specimens was developed. Using this method, the whole genome sequence of HTNV L, M, and S segments from four ROK and US Army military HFRS patients was obtained and compared with genome sequences from natural hosts captured from endemic areas to perform the phylogeographic analysis. Based on this analysis, the most likely site of HTNV infections was determined to be the field military areas where patients had trained during the 50 days previous to the onset of symptoms. In addition, analysis of the HTNV tripartite genomes suggested a recombination of the S segment in nature.

## Results

### Whole genome sequencing of HTNV using multiplex PCR-based NGS

**Figure 1:**
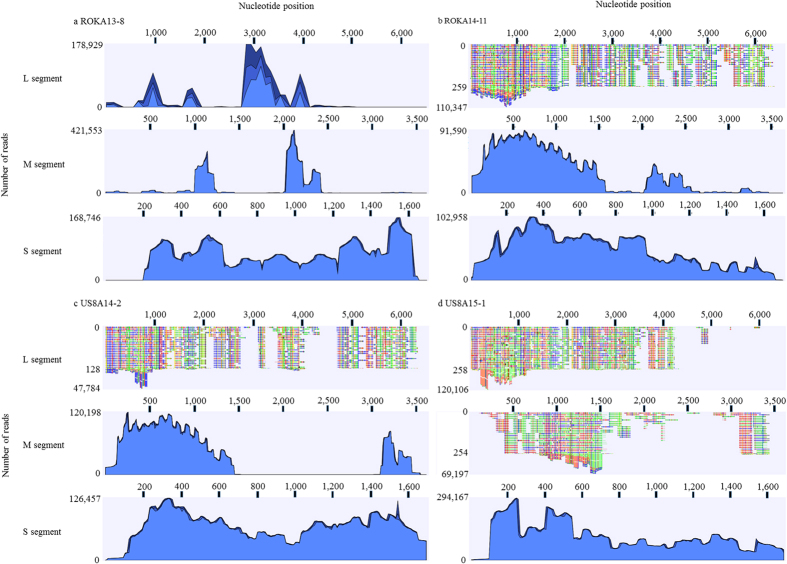
Coverage of HTNV tripartite genomes from HFRS patient samples by multiplex PCR-based NGS. Whole genome sequencing of HTNV was performed by multiplex PCR-based NGS. The number and coverage of reads for HTNV tripartite genomes is shown. The reads were analyzed by reference matching mapping for HTNV 76-118 (L segment, NC005222; M segment, M14627; S segment, M14626). CLC Genomic Workbench version 7.5.2 was used. (**a**) ROKA13-8 (**b**) ROKA14-11 (**c**) US8A14-2 (**d**) US8A15-1.

To obtain whole genome sequence of HTNV from the patient samples, HTNV tripartite genomes were amplified by performing HTNV-specific multiplex PCR. The coverage of HTNV tripartite genomes from ROKA13-8, ROKA14-11, US8A14-2, and US8A15-1 is shown (Fig. 1). The genomic sequence of HTNV L, M, and S segments from ROKA 13-8 was recovered up to 85.9%, 98.2%, and 100% based on full-length of the prototype HTNV 76–118 tripartite genomes, respectively. For ROKA14-11, 97.2% of HTNV L segment was obtained and the consensus sequence of HTNV M and S segments was completely obtained. The coverage of HTNV L segment from US8A14-2 was 96.1% whereas the HTNV M and S segments were completely sequenced. The genomic sequence of HTNV from US8A15-1 was acquired by 87.4%, 98.2%, and 100% for L, M, and S segments, respectively.

### Phylogeographic analyses of HFRS patients

**Figure 2:**
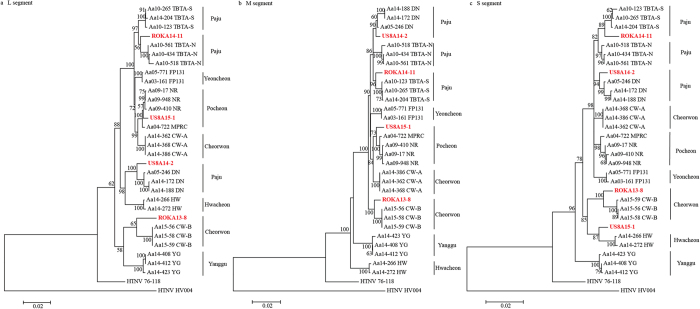
Phylogenetic analysis of four HTNV strains from HFRS patients. Phylogenetic trees were generated by ML method (MEGA 5.2), using the TN92 (Tamura 3-parameter) + G model of evolution, based on the alignment of the tripartite genomes of HTNV. Topologies were evaluated by bootstrap analysis of 1000 iterations. (**a**) HTNV L segment genome sequences (1–4,291nt), (**b**) HTNV M segment genome sequences (1–3,550 nt), (**c**) HTNV S segment genome sequences (1–1,696 nt). Paju, Yangju, Dongducheon, Yeoncheon, and Pocheon are included in Gyeonggi province. Cheorwon, Hwacheon, and Yanggu are included in Gangwon province. Accession number of the sequenced is shown in the supplementary data 2. (TBTA-N, Twin Bridge Training Area North; TBTA-S, Twin Bridge Training Area South; FP 131, Fire Point 131; NR, Nightmare Range; MPRC, Rodriguez Multi-Purpose Range Complex; DN, Dagmar North; HW, Hwacheon; CW-A, Cheorwon-A (Guntan-ri); CW-B, Cheorwon-B (Jigyeong-ri/Munhye-ri); YG, Yanggu).

For phylogeographic analyses, the whole genome sequence of 26 HTNV strains was acquired from rodents collected in endemic areas in the ROK (Fig. 2). The HTNV strains in the phylogenetic tree consisted of 3 strains from Twin Bridge Training Area South (TBTA-S), 3 strains from Twin Bridge Training Area North (TBTA-N), 3 strains from Dagmar North (DN) in Paju, 2 strains from Fire Point 131 (FP131) in Yeoncheon, 4 strains from Nightmare Range (NR) and Rodriguez Multi-Purpose Range Complex (MPRC) in Pocheon in Gyeonggi province, and 6 strains from Cheorwon-A (Guntan-ri) and B (Jigyeong-ri and Munhye-ri), 2 strains from Hwacheon, and 3 strains from Yanggu in Gangwon province. The genomic sequence of HTNV tripartite genomes from *A. agrarius* formed geographic clusters providing a platform for the phylogeographic analysis.

Patient ROKA13-8, assigned to Hwacheon, conducted military training in Cheorwon (Table 1). The Soldier exhibited clinical HFRS symptoms on December 2, 2013, and was hospitalized in the Armed Forces Capital Hospital (AFCH), Armed Forces Medical Command (AFMC) in the ROK (Table 2). Clinical serum was collected from the patient on December 16, 2013. Laboratory diagnosis confirmed HTNV infection by IFA and RT-PCR on February 21, 2014. Phylogenetic analysis demonstrated the L, M, and S segments of ROKA13-8 formed a cluster with the HTNV strains in Cheorwon-B. Patient ROKA14-11, assigned to Paju, conducted military training in Paju (Table 1). The Soldier exhibited clinical HFRS symptoms on November 21, 2014, as shown in Table 2. On December 1, the Soldier was confirmed to be positive for HTNV infection by IFA and RT-PCR. Phylogenetically, the L, M, and S segments of ROKA14-11 associated with the HTNV strains from TBTA-S. Patient US8A14-2 exhibited the onset of clinical symptoms on December 17, 2014 and was admitted to the Brian Allgood Army Community Hospital (BAACH) with diarrhea, vomiting, fever, and rapid pulse on December 21. The Soldier was diagnosed for HFRS while referring also myalgia, headache, vomiting, diarrhea, epigastric pain, petechiae on his thigh, splenomegaly, and kidney inflammation. Laboratory diagnosis confirmed that the Soldier was positive for HTNV by IFA and RT-PCR on December 29. According to the medical record, the Soldier was a humvee driver and tactically moved to Rodriguez MPRC range on October 3, DN on November 17–20, TBTA on November 22–23, Camp Hovey (CH) on November 25–30, and Warrior Base (WB) and Story Range (SR) on December 1–7. Phylogenetic analysis of US8A14-2 demonstrated the L, M, and S segments formed a cluster with HTNV strains at DN. Clinical symptoms of patient US8A15-1 were observed on May 7, 2015, and the Soldier was admitted to the BAACH with diarrhea, vomiting, fever, and rapid pulse on May 16. The clinical specimen was sent to Korea University on May 21 and laboratory diagnosis, using IFA and RT-PCR, confirmed HTNV infection on May 22. The L and M segments of US8A15-1 formed a cluster with HTNV stains in Pocheon. However, phylogenetic analysis of the HTNV S segment from US8A15-1 demonstrated the genetic cluster with HTNV strains in Hwacheon, suggesting a recombination of HTNV S segment genome in nature.

### Recombination analysis of HTNV

**Figure 3:**
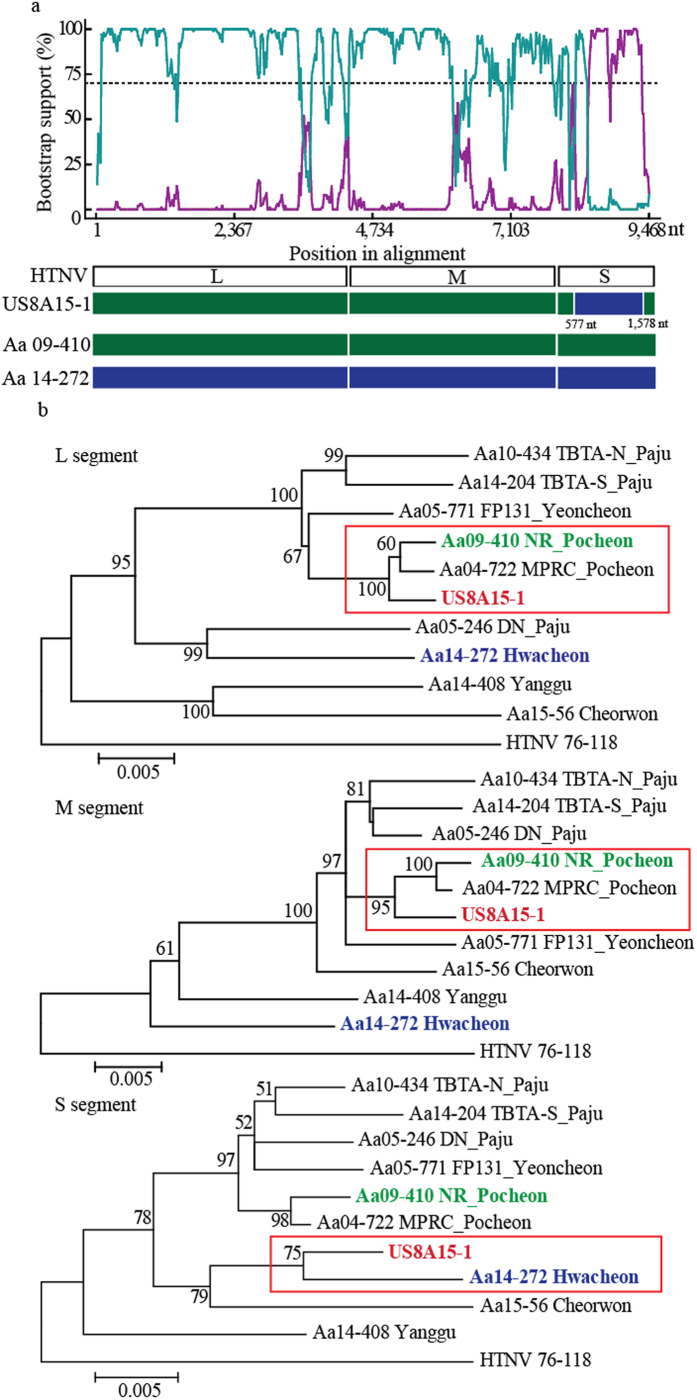
Recombination analysis of US8A15-1 from a HFRS patient. (**a**) The Bootscan plot was based on a pairwise distance model by the RDP4 algorithm. A Bootscan Support Percent of over 70% (cutoff value) was considered significant. Green color represents the comparison of HTNV from US8A15-1 to that from Pocheon. Violet color represents the comparison of HTNV from US8A15-1 to that from Hwacheon. Green bars represent the genome structure of Aa09-410 from Pocheon. Violet bars represent the genome structure of Aa14-272 from Hwacheon. (**b**) The HTNV recombination identified in Gangwon and Gyeonggi provinces was analyzed phylogenetically by the construction of individual ML trees for the L, M, and S segments. Bold red, green, and violet color indicate HTNV US8A15-1, HTNV stains from Pocheon, and Hwacheon, respectively.

To examine the genetic exchange of HTNV S segment, HTNV tripartite genomes were sequentially aligned and analyzed using the RDP4 program (Fig. 3). The L and M segment genomic sequences of US8A15-1 showed high homology with HTNV strains, *Apodemus agrarius* (Aa) 04-722 and Aa09-410 collected from Pocheon. The partial sequence (coordinated to 577–1,578nt) of S segment of US8A15-1 was highly homologous to Aa14-272 identified from Hwacheon. P-value of the analyses was from 5.803E-5 to 7.013E-11 and the RDP recombination consensus score (RDPRCS) of US8A15-1, approximately 0.659, was relatively high when compared to HTNV strains in Pocheon (RDPRCS = 0.011) and Hwacheon (RDPRCS = 0.330). These results indicated that UA8A15-1 may be a recombinant due to the exchange of partial S segment in nature.

## Discussion

In 2009, a previous study showed a phylogeographic link between four US Soldiers diagnosed with HFRS due to exposure at military training sites and *A. agrarius* positive for HTNV based on a partial sequence of HTNV M segment^20^. This analysis provided mechanisms to identify the location where the soldiers most likely acquired HTNV infections and under what conditions. The analysis of 354 bp-partial sequence of HTNV M segment between HFRS patients and rodent hosts may be insufficient to better define the phylogeographic link for future cases because of the low resolution. A recent study demonstrated that whole genome sequencing of Lassa virus enhanced the resolution of phylogenetic analysis for the molecular diversity and genomic characteristic compared to the partial sequences^21^. The extension of HTNV tripartite genomic sequences was limited because of the lack of NGS technology and ultra-low copy of viral RNA in clinical samples. Notably, whole genome sequences of HTNV was obtained from HFRS patient sera by multiplex PCR-based NGS using the primer set specific for HTNV L, M, and S segments. This attempt yielded enhanced levels of viral reads throughout the NGS, demonstrating the complete open reading frame genomic sequences of HTNV S and M segments. The multiplex PCR-based NGS is robust and efficient for acquiring the whole genome sequence of HTNV from HFRS patients as well as wild rodent hosts.

**Figure 4:**
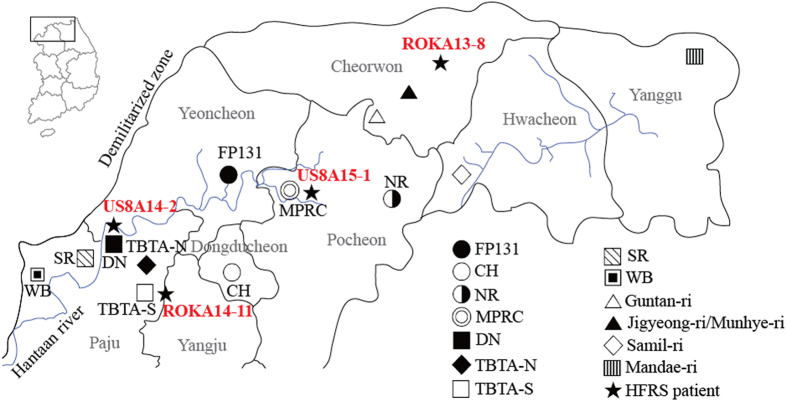
A geographic map of the most likely site of HFRS patients with HTNV infection. The map shows military training sites and places where rodent surveillance was conducted. The areas are near the demilitarized zone, northern Gyeonggi and Gangwon provinces, Republic of Korea; Paju, Yangju, Dongducheon, Yeoncheon, and Pocheon are included in Gyeonggi province. Cheorwon, Hwacheon, and Yanggu are included in Gangwon province. Based on the phylogeographic analysis, most likely sites where four HFRS patients acquired HTNV infections are indicated; ROKA13-8, Cheorwon; ROKA14-11, TBTA-S; US8A14-2, DN; US8A15-1, Pocheon (FP 131, Fire Point 131; CH, Camp Hovey; NR, Nightmare Range; MPRC, Rodriguez Multi-Purpose Range Complex; DN, Dagmar North; TBTA-N, Twin Bridge Training Area North; TBTA-S, Twin Bridge Training Area South; SR, Story Range; WB, Warrior Base). (We used Adobe Illustrator CS6 (http://www.adobe.com/products/illustrator.html) to create the map).

To establish the phylogeographic map, HTNV tripartite genome sequences from *A. agrarius* captured in the endemic and military training areas near the demilitarized zone were completely acquired using RT-PCR. Compared with the genomic HTNV sequences of patients and the rodent hosts, the site where HFRS patients acquired HTNV infection was suggested by the phylogeographic analyses (Fig. 4). ROKA13-8 and ROKA14-11 (Korean army HFRS patients) formed a cluster with HTNV strains from Cheorwon-B and TBTA-S, respectively. Patient ROKA13-8 was assigned to Hwacheon, but conducted military training in Cheorwon. ROKA13-8 was phylogenetically associated with HTNV strains in Cheorwon-B suggesting the Soldier was most likely infected with HTNV during the training in Cheorwon. This result also suggested that patient ROKA14-11 conducted military activity and was the most likely infected with HTNV near TBTA-S in Paju. Phylogenetic analysis of US Army HFRS patient US8A14-2 showed a close relationship with HTNV strains from DN in Paju. This result and clinical records suggested the Soldier was the most likely infected with HTNV at DN between November 17 and 20, fell within the normal incubation period of HTNV. The US Army HFRS patient US8A15-1 conducted military training at MPRC, Pocheon. The phylogenetic association of US8A15-1 with HTNV strains in Pocheon identified the most likely site infection. The phylogenetic and geographic analyses of HTNV whole genome sequences from the patients and natural reservoirs provide an epidemiological diagnostic and surveillance system for annual endemic HTNV infections, identifying the most likely site of infection and under what conditions the infection was acquired. These data contribute to the development of disease risk analyses and preventive measures by changing the environment that reduces rodent habitat and training site avoidance during periods of highest risk^22^.

Recombination is a molecular genetic mechanism that confers the genetic diversity in RNA viruses in nature^23^. The genetic event of bunyaviruses was observed in nature and *in vitro*^24^,^25^. The frequency of the recombination of hantavirus tripartite genomes may be associated with the function of proteins encoded on the L, M, and S segments. Diverse recombinants generated by the exchange of different genetic information could be present. For instance, Seoul virus, Andes virus, Tula virus, and Pummala virus generated the S segment recombinants, whereas the M segment recombination was observed in HTNV, China^26^. In this study, the phylogenetic tree demonstrated L and M segments of US8A15-1 formed clusters with HTNV in Pocheon but the partial sequence of S segment of the HTNV was closely associated to that of HTNV in Hwacheon. This observation suggests that US8A15-1 appears to be an S segment recombination of HTNV with 0.659 of RDPRCS and *P*-value < 0.05. In addition, the exchange of genetic components may be a critical factor in the pathogenicity of the viral infections^27^. Given our limited knowledge of genetic events in nature, whether the S segment recombination is a virulence determinant of hantavirus-associated diseases remains to be investigated.

In conclusion, multiplex PCR-based NGS elicited whole genome sequences of HTNV from HFRS patients. In combination with the whole genomic sequences of HTNV from natural reservoirs, *A. agrarius*, phylogeographic analyses may be useful for genomic-based diagnosis and surveillance of hantavirus-borne outbreaks. The novel platform of NGS-based diagnosis provides a potential tool to develop risk analyses and preventive and therapeutic strategies against emerging viral diseases.

## Methods

### Ethics Statement

Human samples were provided with informed consent. The study was approved and carried out in accordance with the ethical Guidelines for the Korea University Institutional Animal Care and Use Committee (KUIACUC), Korea University. Live trapping of rodents at US military training sites and installations was approved by USFK in accordance with USFK Regulation 40–1 “Prevention, Surveillance, and Treatment of Hemorrhagic Fever with Renal Syndrome”. Rodents were transported to Korea University where they were euthanized by cardiac puncture and tissues collected under isoflurane anesthesia in accordance with procedures approved by KUIACUC (#2010–212) protocol.

### Sample collection

Whole blood or serum samples from HFRS-military patients were provided by the Korea Armed Forces Capital Hospital, Gyeonggi province, and the Brian Allgood Army Community Hospital, Seoul, ROK. Serum was extracted by centrifuging at 4000 rpm at 4 °C for 5 min and then stored at −80 °C. *A. agrarius* specimens were collected at US military training sites and installations near the demilitarized zone, Gyeonggi and Gangwon provinces, ROK, using Sherman collapsible live traps (8 by 9 by 23 cm; H. B. Sherman, Tallahassee, FL, USA). The traps were placed at 4–5 m intervals and collected daily over a 1–3 day period.

### Indirect Immunofluorescence Antibody test

Serum was diluted 1:32 and placed into duplicate wells of acetone-fixed Vero E6 cells infected with HTNV, and then incubated at 37 °C for 30 min. After the wells were washed three times with Phosphate-Buffered Saline (PBS), fluorescein isothiocyanate-conjugated goat antibody to mouse immunoglobulin G (IgG) antibodies (ICN Pharmaceuticals, Laval, Canada) was added to the wells. The wells were incubated at 37 °C for 30 min, washed three times with PBS, and then examined under a fluorescence microscope. The detection of virus-specific fluorescence was indicative of HTNV infection.

### RNA extraction and RT-PCR

*A. agrarius* lung tissues were mechanically homogenized using TissueLyser (MP Biomedicals, Santa Ana, USA), and human sera were added with TRI Reagent Solution (Ambion, Austin, USA). Total RNA was extracted using the Hybrid R^TM^ Kit (GeneAll, Seoul, Republic of Korea) according to the manufacturer’s specifications. cDNA was generated using M-MLV (Promega, Madison, USA) with a random hexamer or OSM55, followed by nested PCR performed in 25 μl reaction mixture containing 250 μM dNTP, 2 mM MgCl_2_, 1U of Super-Therm Taq DNA polymerase (JMR Holdings, London, UK), 1.5 μl of cDNA template and 5 pM of each primer^28^. Initial denaturation was performed at 94 °C for 4 min, followed by 6 cycles of denaturation at 94 °C for 30 sec, annealing at 37 °C for 30 sec, elongation at 72 °C for 1 min, followed by 30 cycles of denaturation at 94 °C for 30 sec, annealing at 42 °C for 30 sec, elongation at 72 °C for 1 min and then elongation at 72 °C for 5 min using ProFlex PCR System (Life technology, Carlsbad, USA).

### Genomic sequencing of HTNV from *A. agrarius*

Viral cDNA from lung tissues of *A. agrarius* was synthesized with random hexamers or OSM55 (5′-TAGTAGTAGACTCC-3′). Conventional nested PCR was performed using Solg^TM^ 2X h-Taq PCR Smart mix (Solgent, Daejeon, Republic of Korea). The PCR program is an initial denaturation at 95 °C for 15 min, followed by 40 cycles of denaturation at 95 °C for 20 s, annealing at 50 °C for 40 s, and elongation at 72 °C for 1 min, and then a cycle of 72 °C for 3 min.

### Multiplex PCR

Multiplex PCR primers were designed using BioEdit Sequence Alignment Editor (Version 7.1.11). Each primer for the multiplex PCR of HTNV L, M, and S segments was prepared and mixed. cDNA synthesized above was amplified twice for HTNV using the primer mixtures and Solg^TM^ 2X Uh-Taq PCR Smart mix (Solgent) according to the manufacturer’s instruction. The first PCR was performed in 25 μl reaction mixture containing 12.5 μl 2X Uh pre-mix, 1.0 μl of cDNA template, 1.0 μl of each primer mixture, and 10.5 μl distilled water (DW). Initial denaturation was performed by a cycle of 95 °C for 15 min, then 40 cycles of 95 °C for 20 sec, 50 °C for 40 sec, 72 °C for 1 min, and a cycle of final elongation at 72 °C for 3 min. The second PCR was performed in 25 μl reaction mixture containing 12.5 μl 2X Uh pre-mix, 1.0 μl of the first PCR product, 1.0 μl of each primer mixture, and 10.5 μl DW. Initial denaturation was performed at 95 °C for 15 min, then 25 cycles of 95 °C for 20 sec, 50 °C for 40 sec, 72 °C for 1 min, and final elongation at 72 °C for 3 min. The primer sequences are shown in supplementary data 1.

### Next generation sequencing

The Multiplex PCR product was prepared using a TrueSeq Nano DNA LT sample preparation kit (Illumina, San Diego, USA) according to the manufacturer’s instructions to generate MiSeq sequencing libraries. The samples were mechanically fragmented using an M220 focused ultrasonicator (Covaris, Woburn, USA). The cDNA amplicon was size-selected, poly-A-tailed, and ligated with indexes and adaptors, followed by the enrichment with 5 μl of PCR primer cocktail and 20 μl of enhanced PCR mixture. Quality control of the libraries was performed using an Agilent DNA 1000 chip kit or High-sensitivity DNA chip kit on a bio-analyzer (Agilent Technologies, Santa Clara, USA). The libraries were quantified by real-time PCR using the Library Quantification Kit for Illumina sequencing platforms (KAPA Biosystems, Wilmington, USA) using a Quantstudio 6 Flex Real-Time PCR System (Life Technologies). The libraries were normalized to a concentration of 12 pM using hybridization buffer. Sequencing was run on a MiSeq benchtop sequencer (Illumina) with 2 × 150 bp using a MiSeq reagent V2 (Illumina). Illumina FASTQ files were imported and analyzed using CLC Genomics Workbench software (Qiagen, Waltham, USA).

### Phylogenetic analysis

The genomic sequence of HTNV L, M, and S segments from lung tissues of *A. agrarius* and patient’s sera was aligned using the Clustal W method with the Lasergene program, version 5 (DNASTAR). Phylogenetic analysis was conducted using the Neighbor-Joining (NJ) and Maximum Likelihood (ML) methods (MEGA 5.2)^29^. Topologies were evaluated by bootstrap analysis of 1000 iterations.

### Recombination analysis

Sequence sets to HTNV L, M, and S segments were aligned and analyzed using the Recombination Detection Program 4 (RDP4) software package^30^. The recombination event evaluated by RDP4 was considered significant if it satisfied at least 2 criteria when the P-value (p) < 0.05 and the RDP recombination consensus score (RDPRCS) was >0.6^31^. When p < 0.05 and the RDPRCS was between 0.4 and 0.6, the recombination event was possible. An RDPRCS < 0.4 with p < 0.05 indicated the rejection of the recombination event. The whole genome sequences of HTNV L, M, and S segments from recombinants, parents, and in- and out- groups were used to generate ML analysis for inferring evolutionary trees in MEGA 5.2, respectively.

## Additional Information

**How to cite this article**: Kim, W.-K. *et al*. Phylogeographic analysis of hemorrhagic fever with renal syndrome patients using multiplex PCR-based next generation sequencing. *Sci. Rep*. **6**, 26017; doi: 10.1038/srep26017 (2016).

## Supplementary Material

Supplementary Table S1

Supplementary Table S2

## Figures and Tables

**Table 1:** Summary of patient sample information.

Patient	Onset	Date of diagnosis	Date of sample Collected	Date of Laboratory diagnosis	Date of outdoor activities	Activity
ROKA13-8	Dec. 2, 2013	Dec. 6, 2013	Dec. 16, 2013	Feb. 21, 2014	–*	Military training
ROKA14-11	Nov. 21, 2014	Nov. 21, 2014	Nov. 28, 2014	Dec. 01, 2014	Nov. 17–21, 2014	Stakeout in the hill
US8A14-2	Dec. 17, 2014	Dec. 21, 2014	Dec. 24, 2014	Dec. 29, 2014	Oct. 3-Dec. 7, 2014	Driving a humvee
US8A15-1	May 7, 2015	May 16, 2015	May 21, 2015	May 22, 2015	–*	Military training

^*^unknown.

**Table 2:** Clinical symptoms of HFRS patients.

Patient	Fever	Chill	Headache	Myalgia	Conjunctival injection	Petechiae	Shortness of breath	Hematuria	Polyuria	Oliguria	Pain
ROKA13-8	Yes	Yes	Yes	Yes	Yes	No	No	Yes	–^*^	Yes	Yes
ROKA14-11	Yes	Yes	Yes	No	Yes	No	No	Yes	Yes	Yes	Yes
US8A14-2	Yes	–*	Yes	Yes	–^*^	Yes	–*	–*	–*	Yes	Yes
US8A15-1	Yes	Yes	Yes (body aches)	–*	–*	No	Yes	–*	–*	Yes	Yes

^*^unknown.

## References

[b1] Radford A. D. . Application of next-generation sequencing technologies in virology. J Gen Virol 93, 1853–1868 (2012). 22647373 10.1099/vir.0.043182-0 PMC3709572

[b2] Gire S. K. . Genomic surveillance elucidates Ebola virus origin and transmission during the 2014 outbreak. Science 345, 1369–1372 (2014). 25214632 10.1126/science.1259657 PMC4431643

[b3] Sharif-Yakan A. & Kanj S. S. Emergence of MERS-CoV in the Middle East: origins, transmission, treatment, and perspectives. PLoS Pathog 10, e1004457 (2014). 25474536 10.1371/journal.ppat.1004457 PMC4256428

[b4] Quinones-Mateu M. E., Avila S., Reyes-Teran G. & Martinez M. A. Deep sequencing: becoming a critical tool in clinical virology. J Clin Virol 61, 9–19 (2014). 24998424 10.1016/j.jcv.2014.06.013 PMC4119849

[b5] Barzon L. . Next-generation sequencing technologies in diagnostic virology. J Clin Virol 58, 346–350 (2013). 23523339 10.1016/j.jcv.2013.03.003

[b6] Guerreiro R., Bras J., Hardy J. & Singleton A. Next generation sequencing techniques in neurological diseases: redefining clinical and molecular associations. Hum Mol Genet 23, R47–53 (2014). 24794858 10.1093/hmg/ddu203 PMC4170717

[b7] Luthra R., Chen H., Roy-Chowdhuri S. & Singh R. R. Next-Generation Sequencing in Clinical Molecular Diagnostics of Cancer: Advantages and Challenges. Cancers 7, 2023–2036 (2015). 26473927 10.3390/cancers7040874 PMC4695874

[b8] Ma Y., Shi N., Li M., Chen F. & Niu H. Applications of Next-generation Sequencing in Systemic Autoimmune Diseases. Genom Proteom Bioinform 13, 242–249 (2015). 10.1016/j.gpb.2015.09.004 PMC4610970 26432094

[b9] Karlsson F., Tremaroli V., Nielsen J. & Backhed F. Assessing the human gut microbiota in metabolic diseases. Diabetes 62, 3341–3349 (2013). 24065795 10.2337/db13-0844 PMC3781439

[b10] Lefterova M. I., Suarez C. J., Banaei N. & Pinsky B. A. Next-Generation Sequencing for Infectious Disease Diagnosis and Management: A Report of the Association for Molecular Pathology. J Mol Diagn 17, 623–634 (2015). 26433313 10.1016/j.jmoldx.2015.07.004

[b11] Luciani F., Bull R. A. & Lloyd A. R. Next generation deep sequencing and vaccine design: today and tomorrow. Trends Biotechnol 30, 443–452 (2012). 22721705 10.1016/j.tibtech.2012.05.005 PMC7127335

[b12] Vaheri A. . Uncovering the mysteries of hantavirus infections. Nat Rev Microbiol 11, 539–550 (2013). 24020072 10.1038/nrmicro3066

[b13] Vapalahti O. . Hantavirus infections in Europe. Lancet Infect Dis 3, 653–661 (2003). 14522264 10.1016/s1473-3099(03)00774-6

[b14] Song J.-W. . Genetic diversity of *Apodemus agrarius*-borne hantaan virus in Korea. Virus Genes 21, 227–232 (2000). 11129640 10.1023/a:1008199800011

[b15] Song J.-W. . Isolation of pathogenic hantavirus from white-footed mouse (*Peromyscus leucopus*). Lancet 344, 1637 (1994). 7984010 10.1016/s0140-6736(94)90430-8

[b16] Jonsson C. B., Figueiredo L. T. & Vapalahti O. A. Global perspective on hantavirus ecology, epidemiology, and disease. Clin Microbiol Rev 23, 412–441 (2010). 20375360 10.1128/CMR.00062-09 PMC2863364

[b17] Lee H. W., Lee P. W. & Johnson K. M. Isolation of the etiologic agent of Korean Hemorrhagic fever. J Infect Dis 137, 298–308 (1978). 24670 10.1093/infdis/137.3.298

[b18] Schmaljohn C. & Hjelle B. Hantaviruses: a global disease problem. Emerg Infect Dis 3, 95–104 (1997). 9204290 10.3201/eid0302.970202 PMC2627612

[b19] Mackow E. R. & Gavrilovskaya I. N. Hantavirus regulation of endothelial cell functions. Thromb Haemost 102, 1030–1041 (2009). 19967132 10.1160/TH09-09-0640

[b20] Song J.-W. . Hemorrhagic fever with renal syndrome in 4 US soldiers, South Korea, 2005. Emerg Infect Dis 15, 1833–1836 (2009). 19891878 10.3201/eid1511.090076 PMC2857219

[b21] Andersen K. G. . Clinical Sequencing Uncovers Origins and Evolution of Lassa Virus. Cell 162, 738–750 (2015). 26276630 10.1016/j.cell.2015.07.020 PMC4537774

[b22] Klein T. A. . Hantaan virus surveillance targeting small mammals at nightmare range, a high elevation military training area, Gyeonggi Province, Republic of Korea. PLoS One 10, e0118483 (2015). 25874643 10.1371/journal.pone.0118483 PMC4398386

[b23] Simon-Loriere E. & Holmes E. C. Why do RNA viruses recombine? Nat Rev Microbiol 9, 617–626 (2011). 21725337 10.1038/nrmicro2614 PMC3324781

[b24] Sibold C. . Recombination in Tula hantavirus evolution: analysis of genetic lineages from Slovakia. J Virol 73, 667–675 (1999). 9847372 10.1128/jvi.73.1.667-675.1999 PMC103873

[b25] Plyusnin A. . Transfection-mediated generation of functionally competent Tula hantavirus with recombinant S RNA segment. EMBO J 21, 1497–1503 (2002). 11889055 10.1093/emboj/21.6.1497 PMC125929

[b26] Han G. Z. & Worobey M. Homologous recombination in negative sense RNA viruses. Viruses 3, 1358–1373 (2011). 21994784 10.3390/v3081358 PMC3185808

[b27] Plyusnina A. & Plyusnin A. Recombinant Tula hantavirus shows reduced fitness but is able to survive in the presence of a parental virus: analysis of consecutive passages in a cell culture. Virol J 2, 12 (2005). 15725355 10.1186/1743-422X-2-12 PMC552329

[b28] Kang H. J. . Host switch during evolution of a genetically distinct hantavirus in the American shrew mole (*Neurotrichus gibbsii*). Virology 388, 8–14 (2009). 19394994 10.1016/j.virol.2009.03.019 PMC2692302

[b29] Tamura K. . MEGA5: molecular evolutionary genetics analysis using maximum likelihood, evolutionary distance, and maximum parsimony methods. Mol Biol Evol 28, 2731–2739 (2011). 21546353 10.1093/molbev/msr121 PMC3203626

[b30] Martin D. P. . RDP4: Detection and analysis of recombination patterns in virus genomes. Virus Evol 1, vev003 (2015). 10.1093/ve/vev003 PMC5014473 27774277

[b31] Zhou Z. . Reassortment and migration analysis of Crimean-Congo haemorrhagic fever virus. J Gen Virol 94, 2536–2548 (2013). 23939975 10.1099/vir.0.056374-0

