# Supplementary material for: Phylogeographic analysis of hemorrhagic fever with renal syndrome patients using multiplex PCR-based next generation sequencing
Source: Sci Rep. 2016 May 25;6:26017. doi: 10.1038/srep26017 (PMC4879520; doi:10.1038/srep26017)
Supplement: Supplementary Table S1 [file srep26017-s1.pdf]

Phylogeographic analysis of hemorrhagic fever with renal syndrome patients using multiplex PCR-based next generation sequencing

Won-Keun Kim, Jeong-Ah Kim, Dong Hyun Song, Daesang Lee, Yong Chul Kim, Sook-Young Lee, Seung-Ho Lee, Jin Sun No, Ji Hye Kim, Jeong Hoon Kho, Se Hun Gu, Seong Tae Jeong, Michael Wiley, Heung-Chul Kim, Terry A. Klein, Gustavo Palacios, and Jin-Won Song

Supplementary Table S1. Multiplex PCR primer lists of HTNV L, M, and S segments

L segment		M segment		S segment	
**Primer**	**Sequence (5' -> 3')**	**Primer**	**Sequence (5' -> 3')**	**Primer**	**Sequence (5' -> 3')**
HNL01F	TAGTAGTAGACTCCGGAAGT	HNM01F	TAGTAGTAGACTCCGCAAAATAAAG	HNS01F	TAGTAGTAGGCTCCCTAAAG
HNL02F	GTAGAGTGTATAGATTATCT	HNM02F	AATGTCTATGACATGAAAAT	HNS02F	GGCAGAAGGTAAGGGATGCA
HNL03F	AGGCAATAGGGAAAGTACTA	HNM03F	CAGCACAGCTGGTGCCTGAG	HNS03F	GGCAAAGATTGATGAGTTAA
HNL04F	CAAGGCCTTTTTCAAGATGA	HNM04F	TCAGTCACAGTCTAGCCAAA	HNS04F	CATCTGAAAGAGAGATCAAT
HNL05F	AGAGAAAAGAAGCTTAAATA	HNM05F	CGCAGTAGAAAATCAATAAC	HNS05F	TTGTTGTCTATCTTACATCC
HNL06F	CATTTAATGTAGTGGCAGTT	HNM06F	TGAAAAGCTGTTTGATTGCA	HNS06F	TCGGATTCGATTTAAGGATG
HNL07F	AGCTGAGATAAGTTACGTTA	HNM07F	CCCAGATCAGAGTGTGGTCA	HNS07F	AAGGCAGAAGAGATTACACC
HNL08F	AGCCAGCCATATTACATACC	HNM08F	AAAATTTTTGAACAGGTTAA	HNS08F	TGATTGGTTTCCTGGCATTG
HNL09F	GAAATTTTTCATTTTTTGAA	HNM09F	CACCAATTTATGTTCCAACA	HNS09F	CCTTGGTGGTCCTGCAACAA
HNL10F	TAGAAACTTCTTACTAATAC	HNM10F	AGAAACTGCAACTTACTCTA	HNS10F	GAAGCAGCTGGCTGTAGCAT
HNL11F	CTGAACTTAATTCCAGATAC	HNM11F	TCTTATTCTTCCCTTAGTAT	HNS11F	TAGCAGGTATTGCTGAGCTT
HNL12F	CACGAATACAATTTAAACAG	HNM12F	GTGCCATACCACTCATGTGG	HNS12F	GCGAAAGAAATCATCATTCT
HNL13F	ACTCAATAATCATAAAAGTG	HNM13F	ATCATGTGAGGCCTTTTCTG	HNS13F	GGAAAGGAGGCTGTGGACAA
HNL14F	AAAATATTGTCTGATTTAGA	HNM14F	AATTTTGTATGTCAGCGAGT	HNS14F	CCAACCAAGAGCCTTTAAAA
HNL15F	TTCAGAATGAGACCCAGAAG	HNM15F	ACACTATAACAAGCTTATTT	HNS15F	TTATCAGGGGAATCAGTATA
HNL16F	TCATTCAGGATTAAAAAGAT	HNM16F	GCTTGTTACATTCTGTTTTG	HNS16F	AGGCTGCCTTAAGTAGCCTT
HNL17F	GGTTCCTTTATTAGGTTCAT	HNM17F	CAAGAAAACAGGTTAAAATC	HNS17F	TACTAACAACAACACTCTAC
HNL18F	GGGTATCCAAAGTCATGAGC	HNM18F	CCTATAAAGAGTTGAAGGCA	HNS01R	CTCATCTGGATCCTTTCCAT
HNL19F	TACAGAAGACCAAGGGCAGT	HNM19F	TTACAAAGTATGCCAAGTTA	HNS02R	CAGTTGCAATCCTATCTGCC
HNL20F	ATTTTTGACAATCTACGTTA	HNM20F	TTTAGATACAAAAGTAGGTG	HNS03R	TTTAAATCCAGCACATTACC
HNL21F	CATTAGAGGTATATATATAT	HNM21F	CTCCTGTCTGGAATGACAAT	HNS04R	TAGAGCTTTCAGAAGTATCG
HNL22F	AACAGTGGACCAGTCGACTG	HNM22F	CCGTAGGAAGCTAACAAACC	HNS05R	GGATACCGTTAACGTCCTCG
HNL23F	ACCTGTTTCTTTTTATTTGA	HNM23F	CACTGGTTTGATGGTCGTCT	HNS06R	AGTCCACAGACTGCTGTTCT
HNL24F	AGGAAGAAAAGTATGGGGAA	HNM24F	AAAGGGATTACCAATATGAG	HNS07R	TTGTTCAATACGATCACTCC
HNL25F	TGCTGTTGAGTTGGCAGCAA	HNM25F	GAAACCAGTTGGCAGTGCTT	HNS08R	CCACTTGCCGCTGCCGTAAG
HNL26F	TTTAGCCAAACAAGAAATAT	HNM26F	AATGACTGTTTTGTATCTAG	HNS09R	ATTGATGATGGTGACTCAAT
HNL27F	ATTTATCAAATTCAAGACAT	HNM27F	GTGGCGGTCTAATATTTAAA	HNS10R	CATGTCCTGCAGGATGGAAA
HNL28F	AGAGGCAGATAGGGGCTTTT	HNM28F	GTTCCCAGGTAGTTTCAGGA	HNS11R	TTGATTGTGTCCTTCTGAGA
HNL29F	GAGTACATATCATATGGAGG	HNM29F	ATTGATTCCTTTCAATCTTT	HNS12R	TCAGGATCCATATCATCCCC
HNL30F	GTAATCATAAATTCATTAGG	HNM30F	TCTTAGTAACAAAAGACATC	HNS13R	TAAAAGGATTAATACATTCA
HNL31F	TACCTCCATGCTACATAATG	HNM31F	AGGGTTTACATTAACATGTC	HNS14R	TTCCCACCCATAAATGTTCC
HNL32F	AAACTAAGGAACTATATTGA	HNM32F	GTAACATTAACAAGAGGACA	HNS15R	AAATGAAATCTACATCCATA
HNL33F	ACTGGCTGCAGGGTAACTTA	HNM33F	TTGGACTCCATGCTGCTGCA	HNS16R	GTAGTTAAGTTGAGGTAGTT
HNL34F	TTGCTTTTTTGAGTTTGCAC	HNM34F	AAAATGTTGGTTTGTTAAAT	HNS17R	GTAGTAGTTTTGCTCCCTAA
HNL35F	CAACAGATTCAAGCAGGCCA	HNM35F	GTTTTACTGAGCATTCTCTG		
HNL36F	TTAAGATCTCACCAAAGAAA	HNM36F	TTTTTATATTCCAGTATAAT		
HNL37F	ACTCTTAGGTTCTCTGTCAG	HNM01R	TCCCCGAAGCTTACTGTGTG		
HNL38F	CCTCAGGTTGCACAACTAGC	HNM02R	TGATTGGTGATTATCCATGC		
HNL39F	AAGTTAAGCATGCTGATACA	HNM03R	CAACTTCAGTAGACACTGTC		
HNL40F	ATTAAAGCGCGCACTTCTAG	HNM04R	TAAGTACTATTGCATGATAG		
HNL41F	TTTCAACATGAGAGGTTAGG	HNM05R	ATAAACTACCTGTACTCTGT		
HNL42F	CAAAATTTTTAGAGTTATGG	HNM06R	CGATATCAAAGATCCCATGC		
HNL43F	TGACCCAAGTATTGTTACAG	HNM07R	GTATCATTGCATGTTGATTC		
HNL44F	GAATGGGTTACCTTCAGGGA	HNM08R	GAATGCTTCCATGGATCTGA		
HNL45F	GTTGTACATTTTCTAAAGAG	HNM09R	GAGGAACTTTTGCATTGGCA		
HNL46F	AACATTCACAGTCAGAGAAA	HNM10R	ACATGTTTGGCTTCTGTTGA		
HNL47F	CTGGATACAGCCAAGTTCCC	HNM11R	ATAATATCCAGGTAAATCAA		
HNL48F	TTATGAAAAGAATTGCACCT	HNM12R	TGGGGGAGGTTATGTTGAAA		
HNL49F	CTGGCTGAAATCAATGTCAC	HNM13R	CCATTGCAGTACACAACGAT		
HNL50F	CAACAGAAAGACTTAGCTGC	HNM14R	AATAGAGTGTGCTACCCCAG		
HNL51F	TCGAGGATAAGACCTTTTCA	HNM15R	TAAATGTGATTGCTGGTATA		
HNL52F	TATAAAGGGTGATTATTCAG	HNM16R	TCAAACTCTTCCTTTATCTT		
HNL53F	ACAAAGCTTTATGAAGGAGA	HNM17R	GCATTGAGACTGGGGGCATG		
HNL54F	CATCAATTACACAGAAGCAT	HNM18R	TTTTCTTTAGATCATCCCTG		
HNL55F	AGTACGGCACATGGTATTGT	HNM19R	AGAAGAAATATCCACATTGT		
HNL56F	CTTAGAACCACACAGGCAAT	HNM20R	GTGCATAGGAACAGAACCCA		
HNL57F	TATTAGCTCATGCGTTTCAT	HNM21R	GTAGATCAATGGATTGTGCT		
HNL58F	AAATCCCATCATTTCATCTG	HNM22R	CCATAACAGTGAAAGGATGT		
HNL59F	ATATCTGTTGATCTCTTTAT	HNM23R	ACAATCTGATGGATTACAAC		
HNL60F	GTACTGTGAGTTCAATGTCA	HNM24R	TCCTGCTATACCTTATTGTG		
HNL61F	TGTGGATTTTGAGAATATAG	HNM25R	ATTGTACCAATTATACAGAC		
HNL62F	GAATCAAAGAAAATGAGGGG	HNM26R	GCCAAATTGACATGTGGATG		
HNL63F	CTGTTAATATTATCTTAATG	HNM27R	AAATAGGAGTAGTAGCAAAG		
HNL64F	GAGCATTGTCCGAGGATGGG	HNM28R	TCATCAGTAAAGTGCATAGT		
HNL65F	GATGTCATTCCTGATTCATT	HNM29R	GCAAGGATTTTCACCAAGGT		
HNL01R	ATGTCATGTCTCACGGCGTA	HNM30R	AAAAGGTAGGACATTCTGTT		
HNL02R	TATAATGTTTGAAGGGACCC	HNM31R	CCACCTTTTCCTGACACCTT		
HNL03R	TTGTTCCATTAATTTTATAA	HNM32R	AGAAATCCCATTGACCTTGT		

HNL04R	TCTTGTTCAATATAAGCTAA	HNM33R	CACTGAATATCCCTGAAATC
HNL05R	TTGTGTAGTAATATTGGAGC	HNM34R	AGCTATGACTTTTTATGCTT
HNL06R	CTCTCTCCTCAGTTCTGATC	HNM35R	GCTACTATTTTTTTTTAGTG
HNL07R	GCTCCTATCGGTTCCATCCC	HNM36R	TAGTAGTAGACTCCGCAAAATGTTA
HNL08R	AAAGCACTCGAACACTGCTG		
HNL09R	CAGGTTTGTAAGATGTGATT		
HNL10R	GCCATGTCATGGATCAGGTA		
HNL11R	AAATGTTCCTGGCTCTCTTA		
HNL12R	GACTCTCTATTTGCGCACCA		
HNL13R	TCTTGAGTTGATGGCTCAGT		
HNL14R	TGTTGTCTTTTTCAACACAT		
HNL15R	TATTGTATGAGTGTAAGGAC		
HNL16R	ACTAAACCAGGTCCTATCCT		
HNL17R	ATTTAATGCTAGCAGCCTAT		
HNL18R	ACACAGATCTAATTGCATAT		
HNL19R	GAGTATAATGATGTCACTGC		
HNL20R	AAGTGCAACTAACAAGCTTT		
HNL21R	TAAATGAAGGGTAAACTCCA		
HNL22R	TCTTCATTCATATTTCCATG		
HNL23R	CCACATCATATATCCATTTT		
HNL24R	ACTTTGTAGCCAGGACTTTA		
HNL25R	TGAACCTGTCCTGACATACC		
HNL26R	CTCCTCATATAATTTTAAGA		
HNL27R	GCCTGCATCTAGTAGGAAGT		
HNL28R	GCCCCTTGGATTGCAAGAAT		
HNL29R	TGCACTAACATACATGAGTT		
HNL30R	CACAGTTCTTTAGCTTATTG		
HNL31R	TTGATGTGTGGGTCAAGGCT		
HNL32R	TGCAACACCGAAAAGCGAAG		
HNL33R	CATAAATGAATAATGCATCG		
HNL34R	ATCTCTGTATTTACACTAAA		
HNL35R	AAATTCAGCATTTGTCGGGG		
HNL36R	AATCGTCAAAATAACCTAAA		
HNL37R	CTTTCAACTTTACTGGTGCA		
HNL38R	CATTGCACCATTTCCTCCCA		
HNL39R	GCATTGATTTCTGTCTCTTG		
HNL40R	CACTGTACTTTCCCAATAAA		
HNL41R	AATATAATCATAAGTTACAT		
HNL42R	GGAGCTGCAAGGGTGACTGC		
HNL43R	TCTGCAAAACTGTTAGCAGC		
HNL44R	AATTCCATTCAAGAAATCTT		
HNL45R	CAGGAATACTATTTTGTATG		
HNL46R	ATTGTCTTTAAGTCAACAGA		
HNL47R	TACCCTTGATTTAGAAGATT		
HNL48R	TATGAGGCTTCACTCTTATA		
HNL49R	CATCTCCAAATTTCTATGCA		
HNL50R	CCCAACCCTACAGAACCGAT		
HNL51R	ATGCTCGCCTGTTGGCTTGT		
HNL52R	TCAAGACCAAAATTGAATGT		
HNL53R	CGAAACCTCATCCATCATGA		
HNL54R	TCCCCCATTCAAATCCAGAC		
HNL55R	TCCTTTATTGTAATCATAAA		
HNL56R	TACTGTTTGATACCTAATAT		
HNL57R	GAAGATTCTTCATAAAATTC		
HNL58R	GGGTTAATCAGCTTAAGTAA		
HNL59R	TAAGCTCATTTCTTCAGACC		
HNL60R	GTAGGAAATGTTCAATATCT		
HNL61R	AGTCTGACAGGCTCAAGTAT		
HNL62R	CAAATTAAATGTTTTAGAGA		
HNL63R	GATTTTCAAACTGATCAATA		
HNL64R	AACAGTACCATGGTATGCCT		
HNL65R	TTAGTAGTAGGTATGCTCCG		
