# Supplementary material for: Phylogeographic analysis of hemorrhagic fever with renal syndrome patients using multiplex PCR-based next generation sequencing
Source: Sci Rep. 2016 May 25;6:26017. doi: 10.1038/srep26017 (PMC4879520; doi:10.1038/srep26017)
Supplement: Supplementary Table S2 [file srep26017-s2.pdf]

# Phylogeographic analysis of hemorrhagic fever with renal syndrome patients using multiplex PCR-based next generation sequencing

Won-Keun Kim, Jeong-Ah Kim, Dong Hyun Song, Daesang Lee, Yong Chul Kim, Sook-Young Lee, Seung-Ho Lee, Jin Sun No, Ji Hye Kim, Jeong Hoon Kho, Se Hun Gu, Seong Tae Jeong, Michael Wiley, Heung-Chul Kim, Terry A. Klein, Gustavo Palacios, and Jin-Won Song

**Supplementary Table S2. Accession number of HTNV strains from patients and rodents** (FP 131, Fire Point 131; NR, Nightmare Range; MPRC, Rodriguez Multi-Purpose Range Complex; DN, Dagmar North; TBTA-N, Twin Bridge Training Area North; TBTA-S, Twin Bridge Training Area South)

| No | Strain | Collection site | Accession number | | |
|---|---|---|---|---|---|
| | | | L segment | M segment | S segment |
| 1 | ROKA13-8 | Cheorwon | KU207198 | KU207202 | KU207206 |
| 2 | ROKA14-11 | Paju (TBTA-S) | KU207199 | KU207203 | KU207207 |
| 3 | US8A14-2 | Paju (DN) | KU207200 | KU207204 | KU207208 |
| 4 | US8A15-1 | Pocheon | KU207201 | KU207205 | KU207209 |
| 5 | Aa03-161 | Yeoncheon (FP131) | KT934956 | KT934990 | KT935024 |
| 6 | Aa05-771 | Yeoncheon (FP131) | KT934963 | KT934997 | KT935031 |
| 7 | Aa05-172 | Paju (DN) | KT934974 | KT935008 | KT935042 |
| 8 | Aa05-188 | Paju (DN) | KT934975 | KT935009 | KT935043 |
| 9 | Aa05-246 | Paju (DN) | KU207175 | KU207183 | KU207191 |
| 10 | Aa04-722 | Pocheon (MPRC) | KU207174 | KU207182 | KU207190 |
| 11 | Aa09-17 | Pocheon (NR) | KU207176 | KU207184 | KU207192 |
| 12 | Aa09-410 | Pocheon (NR) | KU207177 | KU207185 | KU207193 |
| 13 | Aa09-948 | Pocheon (NR) | KT934966 | KT935000 | KT935034 |
| 14 | Aa10-123 | Paju (TBTA-S) | KT934968 | KT935002 | KT935036 |
| 15 | Aa10-265 | Paju (TBTA-S) | KU207178 | KU207186 | KU207194 |
| 16 | Aa14-204 | Paju (TBTA-S) | KT934977 | KT935011 | KT935045 |
| 17 | Aa10-434 | Paju (TBTA N) | KT934970 | KT935004 | KT935038 |
| 18 | Aa10-518 | Paju (TBTA N) | KT934971 | KT935005 | KT935039 |
| 19 | Aa10-561 | Paju (TBTA N) | KT934972 | KT935006 | KT935040 |
| 20 | Aa14-266 | Hwacheon (Samil-ri) | KT934979 | KT935013 | KT935047 |
| 21 | Aa14-272 | Hwacheon (Samil-ri) | KT934980 | KT935014 | KT935048 |
| 22 | Aa14-362 | Cheorwon (Guntan-ri) | KT934981 | KT935015 | KT935049 |

| | | | | | |
|---|---|---|---|---|---|
| 23 | Aa14-368 | Cheorwon (Guntan-ri) | KT934982 | KT935016 | KT935050 |
| 24 | Aa14-386 | Cheorwon (Guntan-ri) | KT934983 | KT935017 | KT935051 |
| 25 | Aa15-56 | Cheorwon (Jigyeong-ri/Munhye-ri) | KU207179 | KU207187 | KU207195 |
| 26 | Aa15-58 | Cheorwon (Jigyeong-ri/Munhye-ri) | KU207180 | KU207188 | KU207196 |
| 27 | Aa15-59 | Cheorwon (Jigyeong-ri/Munhye-ri) | KU207181 | KU207189 | KU207197 |
| 28 | Aa14-408 | Yanggu (Mandae-ri) | KT934986 | KT935020 | KT935054 |
| 29 | Aa14-412 | Yanggu (Mandae-ri) | KT934987 | KT935021 | KT935055 |
| 30 | Aa14-423 | Yanggu (Mandae-ri) | KT934988 | KT935022 | KT935056 |
| 31 | HTNV 76-118 | Uijeongbu | NC005222 | M14627 | M14626 |
| 32 | HTNV HV004 | China | JQ083393 | JQ083394 | JQ083395 |

12
